# Backbone resonance assignment of the BCL6-BTB/POZ domain

**DOI:** 10.1007/s12104-017-9778-z

**Published:** 2017-09-19

**Authors:** Li-Ying Lin, S. E. Evans, L. Fairall, John W. R. Schwabe, Simon D. Wagner, Frederick W. Muskett

**Affiliations:** 1 Leicester Drug Discovery and Diagnostics Centre, Maurice Shock Building, University of Leicester, University Road, Leicester, LE1 7RH UK; 2 Leicester Institute of Structural and Chemical Biology, Department of Molecular and Cell Biology, University of Leicester, Henry Wellcome Building, University Road, Leicester, LE1 7RN UK; 3 Department of Cancer Studies and Ernest and Helen Scott Haematological Research Institute, University of Leicester, Lancaster Road, Leicester, LE1 7HB UK

**Keywords:** BCL6-BTB/POZ Domain, NMR resonance assignments, Secondary structure

## Abstract

BCL6 is a transcriptional repressor. Two domains of the protein, the N-terminal BTB-POZ domain and the RD2 domain are responsible for recruitment of co-repressor molecules and histone deacetylases. The BTB-POZ domain is found in a large and diverse range of proteins that play important roles in development, homeostasis and neoplasia. Crystal structures of several BTB-POZ domains, including BCL6 have been determined. The BTB-POZ domain of BCL6 not only mediates dimerisation but is also responsible for recruitment of co-repressors such as SMRT, NCOR and BCOR. Interestingly both SMRT and BCOR bind to the same site within the BCL6 BTB-POZ domain despite having very different primary sequences. Since both peptides and small molecules have been shown to bind to the co-repressor binding site it would suggest that the BTB_POZ domain is a suitable target for drug discovery. Here we report near complete backbone ^15^N, ^13^C and ^1^H assignments for the BTB-POZ domain of BCL6 to assist in the analysis of binding modes for small molecules.

## Biological context

BCL6 is a zinc finger transcription factor and regulator of lymphocyte differentiation. The enforced expression of BCL6 prevents terminal B-cell differentiation to plasma cells in vitro and in vivo is sufficient to promote lymphomagenesis in mice (Cattoretti et al. 2005). On the other hand, mice bearing homozygous disruptions of the BCL6 locus do not have germinal centres and are unable to produce high affinity antibodies in response to immunisation. More recently BCL6 has been shown to be essential for the differentiation of the follicular helper CD4 T-cell subset.

BCL6 is also important as a prognostic marker in high grade B-cell lymphoma. The presence of the t(3;14) translocation was associated with improved prognosis. Gene expression studies have shown that relatively high BCL6 mRNA expression is a feature of the germinal centre (GC) type of high grade B-cell lymphoma, and is again associated with good prognosis, whereas the activated B-cell (ABC) type of lymphoma has relatively low BCL6 expression and poorer clinical outlook.

BCL6 is a transcriptional repressor and the N-terminal BTB-POZ domain is responsible for the recruitment of co-repressor molecules and histone deacetylases (Dhordain et al. 1997). The BTB-POZ domain is found in a large family of proteins and crystal structures of several members, including BCL6 have been produced. The BTB-POZ domain mediates dimerisation but is also responsible for recruitment of co-repressors. The binding of co-repressors, SMRT, NCOR and BCOR, have been studied in detail. Interestingly both SMRT and BCOR bind to the same site within the BCL6 BTB-POZ domain despite having different primary sequences (Ahmad et al. 2003; Ghetu et al. 2008).

Peptide or small molecule binders to the co-repressor binding site have been shown to abrogate the effects of BCL6 in normal B-cells. There are also suggestions that these agents can specifically cause apoptosis of BCL6 dependent B-cell lines and primary human B-cell lymphomas and this site on the BTB-POZ domain is, therefore, a validated target for drug discovery.

## Methods and experiments

### Expression and purification

The BCL6-BTB/POZ domain (residues 7–128, with the following mutations C8Q, C67R and C84N) was cloned into an expression vector (as described previously (Evans et al. 2014)), and the recombinant protein was over expressed in *E. coli* strain BL21 (DE3) Rosetta. Uniformly ^15^N, ^13^C/^15^N and ^2^H/^13^C/^15^N labelled samples were grown in modified Spizizen’s media with 1.0 g/L of ^15^N ammonium chloride and 4 g/L of ^13^C-glucose (99%, Sigma Aldrich) as the sole nitrogen and carbon sources. Bacterial cultures were grown at 37 °C to an optical density of ~ 0.5, whereupon the temperature of the culture was reduce to 20 °C and protein expression was induced by addition of IPTG to a final concentration of 0.225 mM. Cultures were grown for a further 16 h (^15^N, ^13^C/^15^N labelling) or 40 h (^2^H/^13^C/^15^N labelling). Cells were harvested and then lysed into 50 mM Tris, pH 8.5. Initial purification (~70%) was achieved by affinity chromatography using Ni–NTA resin. After TEV cleavage of the affinity tag and dialysis into 50 mM phosphate buffer pH 6.0, 300 mM NaCl, 1 mM DTT, the protein was further purified by size exclusion chromatography using a Superdex S200 column (GE Lifesciences). The protein was concentrated and its purity assessed by Coomassie stained SDS–PAGE.

Two NMR samples were prepared, referred to as “low-salt” and “high-salt”. For the “low-salt” samples, the protein was dialysed against 50 mM sodium phosphate pH 6.8, 50 mM L-arginine, 50 mM L-glutamic acid, 1 mM TCEP, 3 mM sodium azide and 5% v/v D_2_O (Golovanov et al. 2004) and then concentrated to ~200 μM dimer. For the “high-salt” samples, the protein was dialysed against 50 mM sodium phosphate pH 6.8, 300 mM NaCl, 1 mM TCEP, 3 mM sodium azide and 5% v/v D_2_O and then concentrated to ~200 μM dimer. In these buffer conditions the protein remained stable for several weeks.

### NMR chemical shift assignment

NMR spectra were acquired from 350 µl of “low-salt” samples in 5 mm Shigemi NMR tubes, or 120 µl of “high-salt” samples in 3 mm Shigemi NMR tubes. All NMR data were acquired at 25 °C on either 600 or 800 MHz Bruker Avance II/III systems with cryogenically cooled probeheads. The 2D and 3D spectra recorded on the “low-salt” samples to obtain sequence specific assignments were in-house versions of: ^15^N/^1^H TROSY, and trosy versions of HNCACB, HN(CO)CACB, HN(CO)CA, HNCA [reviewed in (Cavanagh 2007)]. Typical acquisition times in F_1_ and F_2_ for the 3D experiments were 17–24 ms for ^15^N, ~9.0 ms for ^13^C, and with an acquisition time of 70 ms in F_3_ (^1^H). Typical acquisition times in 2D experiments were 60 ms (^15^N) in F_1_ and 70 ms in F_2_(^1^H). The WATERGATE method was used to suppress the water signal. Assignments were transferred to the “high-salt” samples by recording an additional HNCACB and HN(CO)CA spectra.

All NMR data sets were non-uniform sampled to 20–25% of the full data size, and were then reconstructed and processed using hmsIST (Hyberts et al. 2012) in conjunction with NMRPipe (Delaglio et al. 1995), and analysed using the Sparky package (T.D. Goddard and D.G. Kneller, Sparky 3, University of California, San Francisco). Sequence-specific backbone resonance assignments (N, NH, Cα, and Cβ) were obtained from the identification of intra- and inter-residue connectivities in HNCACB, HN(CO)CACB, HN(CO)CA and HNCA.

**Fig. 1:**
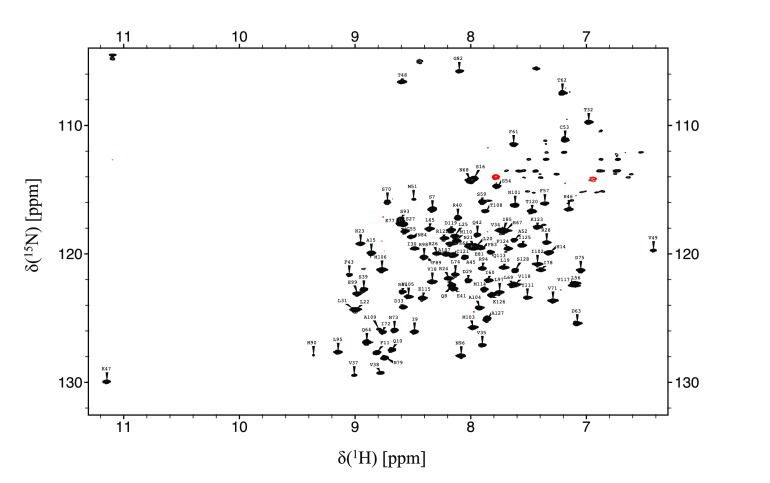
^15^N-TROSY spectrum of the BCL6-BTB/POZ domain. Residue type and number indicate the assignments of the resonances from the backbone amide groups. The spectrum was collected at 25 °C on a Bruker 800 MHz Avance II spectrometer fitted with a cryoprobe. Resonances in green are arginine Hε /Nε signals aliased from ~75 ppm (^15^N)

**Fig. 2:**
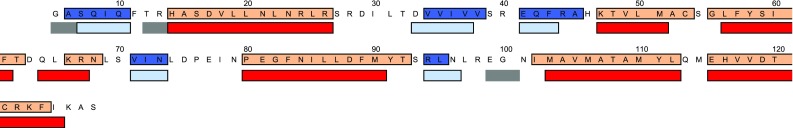
Location of the secondary structural elements in the BCL6-BTB/POZ domain. Highlighted in blue and orange on the amino acid sequence are the positions of the sheet and helical regions (respectively) observed in the crystal structure. Indicated underneath the sequence are the regions suggested to be sheet (cyan) and helix (red) by the program Talos+. Residues for which no data could be obtained (due to lack of chemical shift data), are highlighted in grey

The BCL6-BTB/POZ domain gives rise to well-resolved spectra, as illustrated by the ^15^N/^1^H HSQC spectrum shown in Fig. 1. This allowed essentially complete backbone resonance assignments to be made which was used to map the secondary structure. The data shows that the BCL6-BTB/POZ domain contains seven helices and five β-sheets (Fig. 2).

### Extent of assignments and data deposition

Backbone amide resonance assignments were obtained for all residues in the N-terminal domain except for T12, R13, I36, 86, 87, 88, 91, T92, G100, H116 (92%) and for all Cα and Cβ resonances apart from T12, M90, 91, E99 T108 (96%). The comprehensive ^13^C, ^15^N and HN resonance assignments obtained for the BCL6-BTB/POZ domain have been deposited at the BioMagResBank database. (Accession Number 27,079). Chemical shift differences in the ^15^N-TROSY experiments recorded in the two buffer systems were relatively minor (i.e. less than half a line width) with the following exceptions: V18, R40, Q64, E99, E115, I125, K126 & A127 which showed significant chemical shift changes between “high” and “low” salt buffer systems.

Analysis of the backbone chemical shifts of the BCL6-BTB/POZ domain using Talos+ (Shen et al. 2009) are shown in Fig. 2. These results are in close agreement with the secondary structural elements in the crystal structure (PDB Accession Code: 4cp3 (Evans et al. 2014).
